# Comparison of survival outcomes between squamous cell carcinoma and adenocarcinoma/adenosquamous carcinoma of the cervix after radical radiotherapy and chemotherapy

**DOI:** 10.1186/s12885-022-09401-x

**Published:** 2022-03-25

**Authors:** Ping Liu, Mei Ji, Yanxiang Kong, Zhifeng Huo, Qiubo Lv, Qinghuang Xie, Danbo Wang, Biliang Chen, Hailin Wang, Zhumei Cui, Qianqing Wang, Xiaonong Bin, Jinghe Lang, Chunlin Chen

**Affiliations:** 1 Department of Obstetrics and Gynecology, Nanfang Hospital, Southern Medical University, Guangzhou, 510515 China; 2 Department of Obstetrics and Gynecology, The First Affiliated Hospital of Zhengzhou University, Zhengzhou, China; 3 Department of Reproductive Medicine, The Seventh Affiliated Hospital Sun Yat-Sen University, Shenzhen, China; 4 Department of Obstetrics and Gynecology, Beijing Hospital, National Center of Gerontology, Beijing, 100730 China; 5 Foshan Maternal and Child Health Hospital, Foshan, 528000 China; 6 Department of Gynaecology, Liaoning Cancer Hospital, Liaoning Shenyang, 110042 China; 7 Department of Obstetrics and Gynecology, Xijing Hospital of Airforce Medical University, Xi’An, China; 8 Department of Obstetrics and Gynecology, Gansu Provincial Hospital, Lanzhou, China; 9 Department of Gynaecology, The Affiliated Hospital of Qingdao University, Qingdao, 266061 China; 10 Gynecologic Oncology Department, Xinxiang City Central Hospital in Henan Province, Xinxiang, Henan China; 11 Department of Epidemiology, College of Public Health, Guangzhou Medical University, Guangzhou, China; 12 Department of Obstetrics and Gynecology, Peking Union Medical College, Beijing, 100730 China

**Keywords:** Cervical cancer, Squamous cell carcinoma, Adenocarcinoma, Adenosquamous carcinoma, Radiochemotherapy, Overall survival, Disease-free survival

## Abstract

**Background:**

This study aimed to compare the survival outcomes between squamous cell carcinoma (SCC) and adenocarcinoma/adenosquamous carcinoma (AC/ASC) of the cervix after radical radiotherapy and chemotherapy.

**Methods:**

Propensity score matching (1:4) was used to compare overall survival (OS) and disease-free survival (DFS) in cervical cancer patients with SCC and AC/ASC in China.

**Results:**

Five thousand four hundred sixty-six patients were enrolled according to the criteria. The 5-year OS and DFS in the SCC group (*n* = 5251) were higher than those in the AC/ASC group (*n* = 215). After PSM (1:4), the 5-year OS and DFS in the SCC group were higher than those in the AC/ASC group (72.2% vs 56.9%, *p* < 0.001, HR = 1.895; 67.6% vs 47.8%, *p* < 0.001, HR = 2.056). In stage I-IIA2 patients, after PSM (1:4), there was no significant difference in 5-year OS between the SCC group (*n* = 143) and the AC/ASC group (*n* = 34) (68.5% vs 67.8%, *P* = 0.175). However, the 5-year DFS in the SCC group was higher than that in the AC/ASC group (71.0% vs 55.7%, *P* = 0.045; HR = 2.037, *P* = 0.033). In stage IIB-IV patients, after PSM (1:4), the 5-year OS and DFS in the SCC group (*n* = 690) were higher than those in the AC/ASC group (*n* = 173) (70.7% vs 54.3% *P* < 0.001 vs 1.940%, *P* < 0.001 vs 45.8%, *p* < 0.001).

**Conclusions:**

For stage I-IIA2, there was no significant difference in 5-year survival time, but patients with AC/ASC were more likely to relapse. In the more advanced IIB-IV stage, the oncological outcome of radical radiotherapy and chemotherapy of cervical AC/ASC was worse than that of SCC.

## Background

Cervical cancer is the fourth leading cause of cancer death among women worldwide. Squamous cell carcinoma (SCC) is the most common pathological type, followed by adenocarcinoma and adenosquamous carcinoma (AC/ASC). In 2018, there were 13,240 new cases in the United States, with 4170 deaths [1]. SCC accounts for approximately 70% of cervical cancer, and AC accounts for approximately 20% [2]. In our Chinese cervical cancer diagnosis and treatment database, there were 63,926 cases of cervical cancer from 2004 to 2018, including 56,141 cases of SCC (87.82%) and 5414 cases of AC/ASC (8.47%).

The current NCCN guidelines recommend referring to SCC for the clinical treatment of AC [3], but whether there is a difference in survival outcome between cervical AC/ASC and SCC has been controversial. Most studies suggest that the overall survival rate of patients with early cervical AC/ASC after surgical treatment is lower than that of patients with SCC [4–6], while some think that the survival rates is similar [7, 8]. The oncological outcome of patients with cervical SCC and AC/ASC after radical radiotherapy and chemotherapy is controversial at present. A study based on the National Cancer Database of Korea shows that the survival rate of patients with cervical AC is lower than that of patients with SCC [9]. Other studies also suggest that the oncological outcome of patients with cervical AC/ASC is worse than that of patients with SCC [10–12]. However, Rose PG et al. [13] thought that the prognosis of AC/ASC after simultaneous radiotherapy and chemotherapy was similar to that of SCC, and Katanyoo K et al. [14] thought that the pathological type did not affect the survival outcome.

Therefore, for cervical cancer patients receiving radical radiotherapy and chemotherapy, the prognostic significance of SCC and AC/ASC is worthy of further study, and the above studies lack data from developing countries. China has a large amount of clinical data on cervical cancer, which has important reference value. We combined the Chinese mainland with 37 hospitals that can independently carry out surgical treatment of cervical cancer to conduct a real-world study (RWS) to build a large database on the clinical diagnosis and treatment of cervical cancer in China. Among them, there were 11,433 cases of radical radiotherapy and chemotherapy from 2004 to 2018 and 10,426 cases of SCC (91.19%). AC/ASC accounted for 521 cases (4.56%). The purpose of this study was to further explore the oncological outcome of radical radiotherapy and chemotherapy for cervical SCC and AC/ASC with the help of this large database.

## Methods

### Collection, management, follow-up and storage of the China Cervical Cancer Clinical Database

This retrospective cohort study was conducted following the ethical standards adopted in the 1964 Declaration of Helsinki. Clinical diagnosis and treatment for cervical cancer in China (Four C) was approved by the Ethics Committee of the Nanfang Hospital of Southern Medical University (approval number NFEC-2017–135 and clinical trial number CHiCTR1800017778; International Clinical Trials Registry Platform Search Port, http://apps.who.int/trialsearch/). Uniformly trained gynaecologists collected the cervical cancer patients’ general data, disease-related examination results, adjuvant treatment data and follow-up data from 2004 to 2018.The clinical staging from 2004 to 2009 were revised according to the 2009 FIGO guidelines while cases in 2018 were staged according to FIGO 2018 [15–20].

Trained gynaecologists and monitored by specified staff conducted follow-up phone calls to know the information of patients’ survival, recurrence status,complications and so on. We conducted a thorough search of the outpatient system, picture archiving and communication system (PACS), and clinical laboratory information system if a patient could not be found by telephone. The latest records were considered the time to survival.Two specially trained gynaecologists double-entered the same medical record and then professionals create a unified database of patient data.

### Inclusion and exclusion criteria

Inclusion criteria: Age ≥ 18 years old; pathological diagnosis of cervical cancer by biopsy; the histological type was SCC, AC/ASC; FIGO stage I-IV stage; the year of diagnosis was from 2004 to 2018; initial treatment with radiotherapy(RT),concurrent chemoradiotherapy (CCRT) or radiotherapy + chemotherapy; radiation treatment including external irradiation + afterloading; radiotherapy dose higher than 40 Gy; chemotherapy regimens including paclitaxel + carboplatin, paclitaxel + other platinum, platinum + 5FU, platinum + other, etc., used according to guidelines and drug instructions; survival outcome information available; and all patients able to complete the treatment.

Exclusion criteria: Do not meet the above conditions; special types of cervical cancer: histological types other than SCC or AC/ASC, including undifferentiated carcinoma, neuroendocrine carcinoma, sarcoma, lymphoma and other rare histological types; pregnancy complicated with cervical cancer, accidental discovery of cervical cancer, stump cancer or other malignant tumours; radiotherapy regimen unknown.

### Outcome evaluation and statistical analysis

The primary outcomes were 5 year overall survival (OS) and disease-free survival (DFS).We used SPSS statistical software (version 23.0, SPSS Inc., Chicago, IL, USA) to do the data analysis. Two independent sample t tests were used for continuous variables, and the X2 test or nonparametric test was used for categorical variables or grade variables. In addition, Propensity score matching (1:4) was used according to the patient's age, FIGO stage, histological type, and tumour diameter to adjust the baseline data in this study. The log-rank test in the Kaplan–Meier (KM) method were performed to compare the differences in the survival curves.The hazard ratio was calculated only for the variables included in the Cox regression model. *P* < 0.05 was considered significant.We invited statistical experts to review all statistical methods and procedures used in this study.

## Results

### The screening process of data

**Fig. 1:**
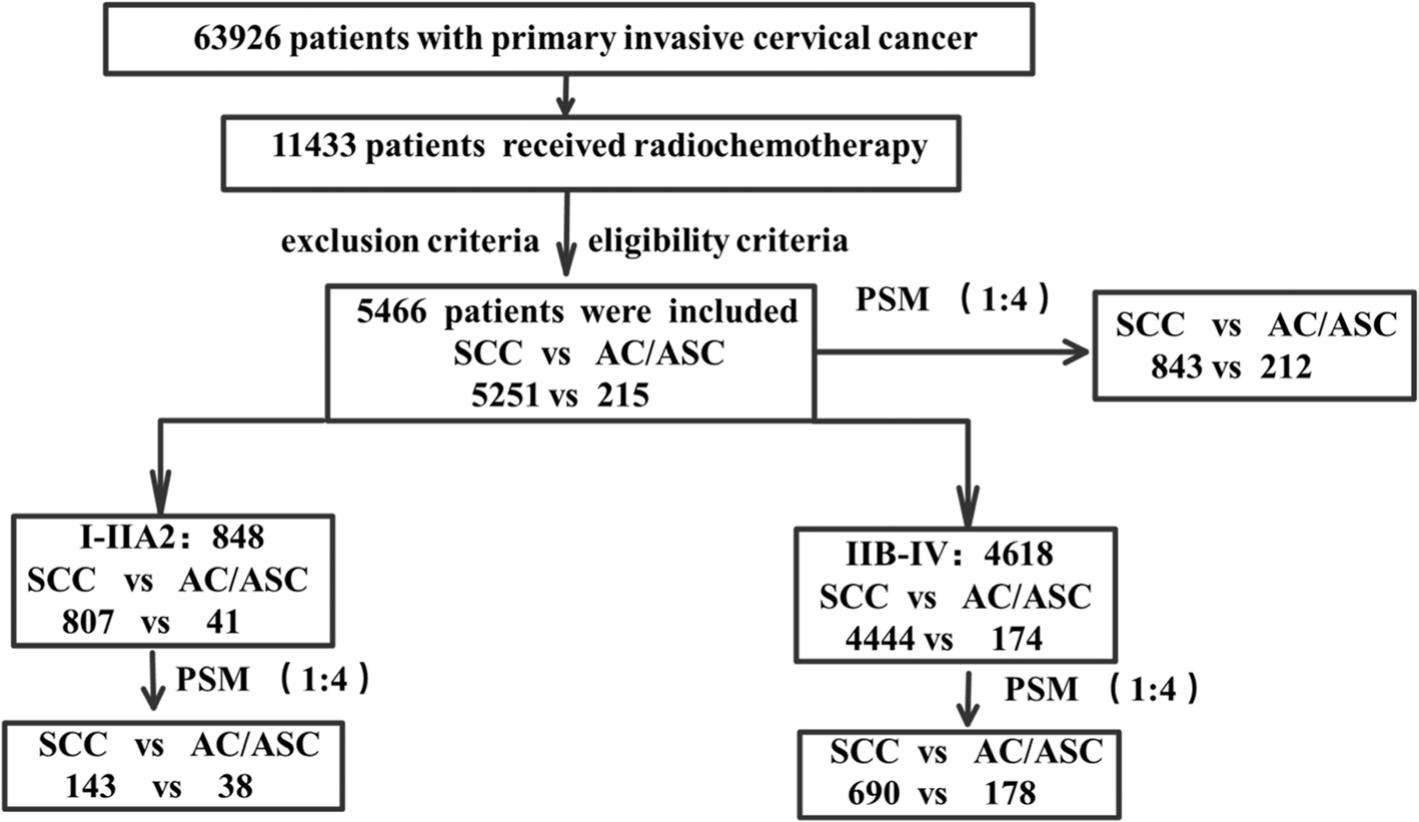
HYPERLINK "sps:id::fig1||locator::gr1||MediaObject::0" Data screening process, *SCC* squamous cell carcinoma, *AC/ASC* adenocarcinoma/adenosquamous carcinoma

Among the 63,926 cases collected by Four C, there were 11,433 patients who underwent radiotherapy and chemotherapy, and 5466 of them met the criteria (SCC vs AC/ASC group, 5251 vs 215). After propensity score matching (1:4), the two groups included 843 and 212 cases, respectively. A total of 848 patients who underwent radiotherapy and chemotherapy in stage I-IIA2 were divided into two groups (SCC vs AC/ASC group, 807 vs 41). After propensity score matching (1:4), the two groups included 143 and 38 cases, respectively. A total of 4618 patients who underwent intermediate radiotherapy and chemotherapy were in stage IIB-IV stage (SCC vs AC/ASC group, 4444 vs 174). After propensity score matching (1:4), the two groups included 690 and 178 cases, respectively. The data screening process is shown in Fig. 1.

### Differences in survival outcomes between the SCC group and AC/ASC group before and after matching: patients meeting the inclusion criteria

To reduce the influence of confounding factors, we performed propensity score matching (1:4) and then survival analysis for the 5466 patients who met the inclusion criteria. Before matching, the median follow-up times for the SCC group and AC/ASC group were 31 months and 26 months, respectively. The number of deaths within 5 years was 998 (19%) and 68 (31.6%), the 5-year OS was 73.0% vs 57.4% (*p* < 0.001), and the DFS was 68.0% vs 48.1% (*p* < 0.001). Cox multivariate analysis showed that the risk of death or recurrence/death was higher in the AC/ASC group (HR = 1.971, *p* < 0.001; HR = 2.106, *P* < 0.001).

After matching, the median follow-up period was 31 months and 26 months. The number of deaths within 5 years was 165 (19.57%) and 68 (32.07%), respectively, and the 5-year OS was 72.2% vs 56.9% (*p* < 0.001). The DFS was 67.6% vs 47.8% (*p* < 0.001).

**Fig. 2:**
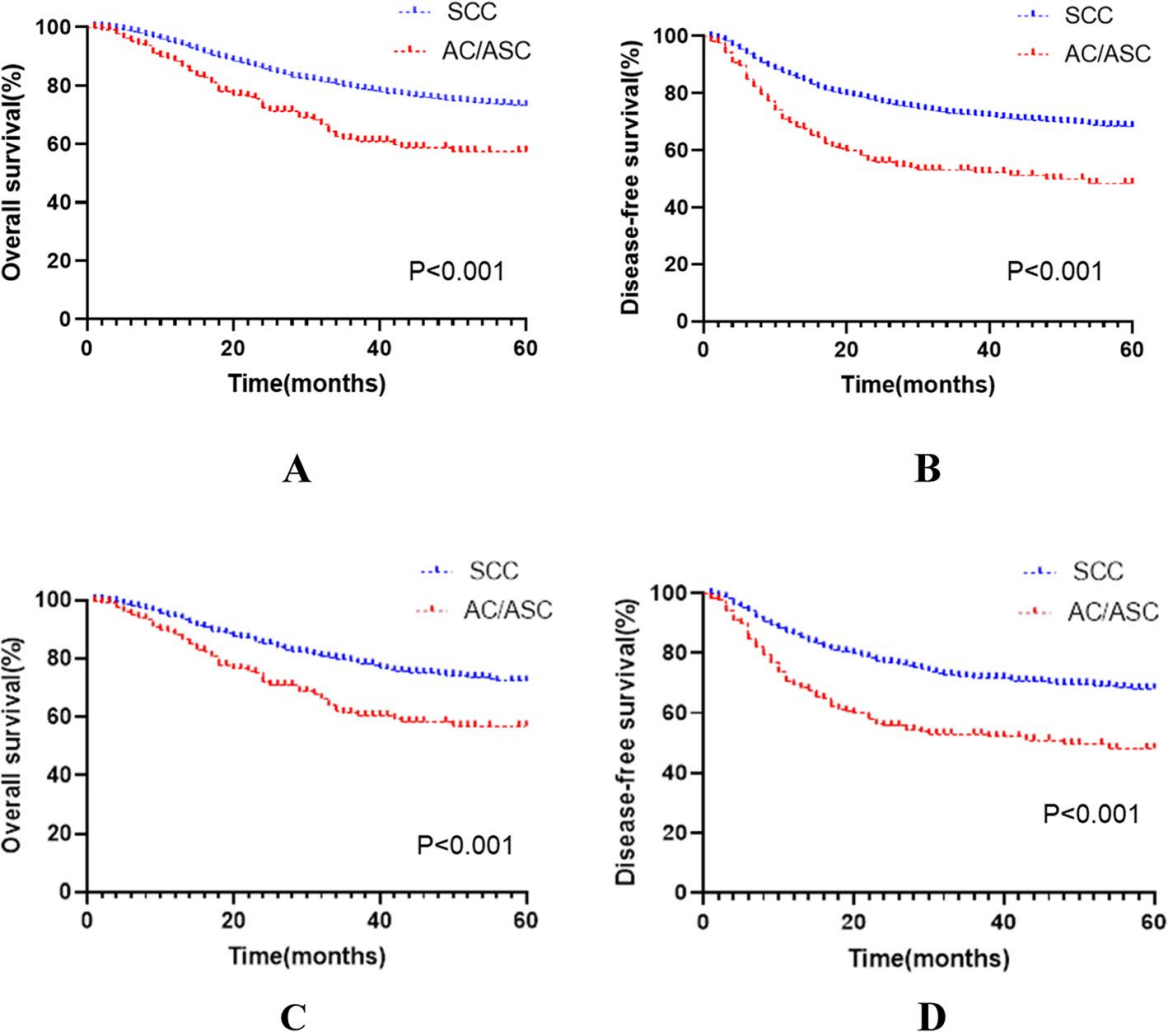
Survival curves before and after patient matching. *Before matching, panels A and B; after matching, panels C and D. *SCC* squamous cell carcinoma, *AC/ASC* adenocarcinoma/adenosquamous carcinoma

Cox multivariate analysis showed that the risk of death or recurrence/death was higher in the AC/ASC group (HR = 1.895, *p* < 0.001; HR = 2.054, *P* < 0.001) (Table 1, Fig. 2).

**Table 1:** Data on all the patients before and after matching

Variables	Unmatched			Matched		
	**SCC (*n* = 5251)**	**AC/ASC (*n* = 215)**	**p-value**	**SCC (*n* = 843)**	**AC/ASC (*n* = 212)**	**p-value**
Age (years)	55.76 ± 10.81	54.45 ± 11.32	0.083	54.66 ± 11.65	54.84 ± 11.50	0.842
FIGO stage			0.003			0.052
I	21 (0.4%)	1 (0.4%)		4 (0.5%)	1 (0.5%)	
II	8 (0.1%)	1 (0.4%)		3 (0.3%)	1 (0.5%)	
IB1	101 (1.9%)	6 (2.8%)		16 (1.9%)	6 (2.8%)	
IB2	79 (1.5%)	6 (2.8%)		15 (1.8%)	6 (2.8%)	
IIA	108 (2.1%)	3 (1.4%)		18 (2.1%)	3 (1.4%)	
IIA1	255 (4.9%)	10 (4.7%)		52 (6.2%)	9 (4.3%)	
IIA2	235 (4.5%)	14 (6.5%)		27 (3.2%)	13 (6.1%)	
IIB	1845 (35.1%)	67 (31.2%)		293 (34.8%)	67 (31.6%)	
III	34 (0.6%)	4 (1.9%)		2 (0.2%)	4 (1.9%)	
IIIA	228 (4.3%)	6 (2.8%)		31 (3.7%)	6 (2.8%)	
IIIB	2142 (40.8%)	78 (36.3%)		343 (40.7%)	78 (36.8%)	
IV	61 (1.2%)	3 (1.4%)		15 (1.8%)	3 (1.4%)	
IVA	31 (0.6%)	4 (1.8%)		6 (0.7%)	4 (1.9%)	
IVB	103 (2.0%)	12 (5.6%)		18 (2.1%)	11 (5.2%)	
Tumour size			0.751			0.988
> 4 cm	2406 (45.8%)	96 (44.5%)		378 (44.8%)	96 (45.3%)	
≤ 4 cm	1754 (33.4%)	77 (35.8%)		299 (35.5%)	74 (34.9%)	
Unknown	1091 (20.8%)	42 (19.5%)		166 (19.7%)	42 (19.8%)	

*FIGO* International Federation of Gynecology and Obstetrics, *SCC* squamous cell carcinoma, *AC/ASC* adenocarcinoma/adenosquamous carcinoma

### Differences in survival outcomes between the two groups before and after matching: patients with stage I-IIA2 cervical cancer

In stage I-IIA2, the baseline of the SCC group (*n* = 807) and AC/ASC group (*n* = 41) was unbalanced. After propensity score matching (1:4), 143 cases and 38 cases were included in each group, and there was no significant difference in survival analysis between the two groups. Before matching, the median follow-up period was 32 months and 27 months. The number of deaths within 5 years was 126 (15.61%) and 9 (21.95%), the 5-year OS was 76.1% vs 70.5% (*P* < 0.01), and the DFS was 72.8% vs 56.8% (*P* = 0.015). Cox multivariate analysis showed that there was no significant difference in the risk of death between the two groups, but the risk of recurrence/death was higher in the AC/ASC group.

**Fig. 3:**
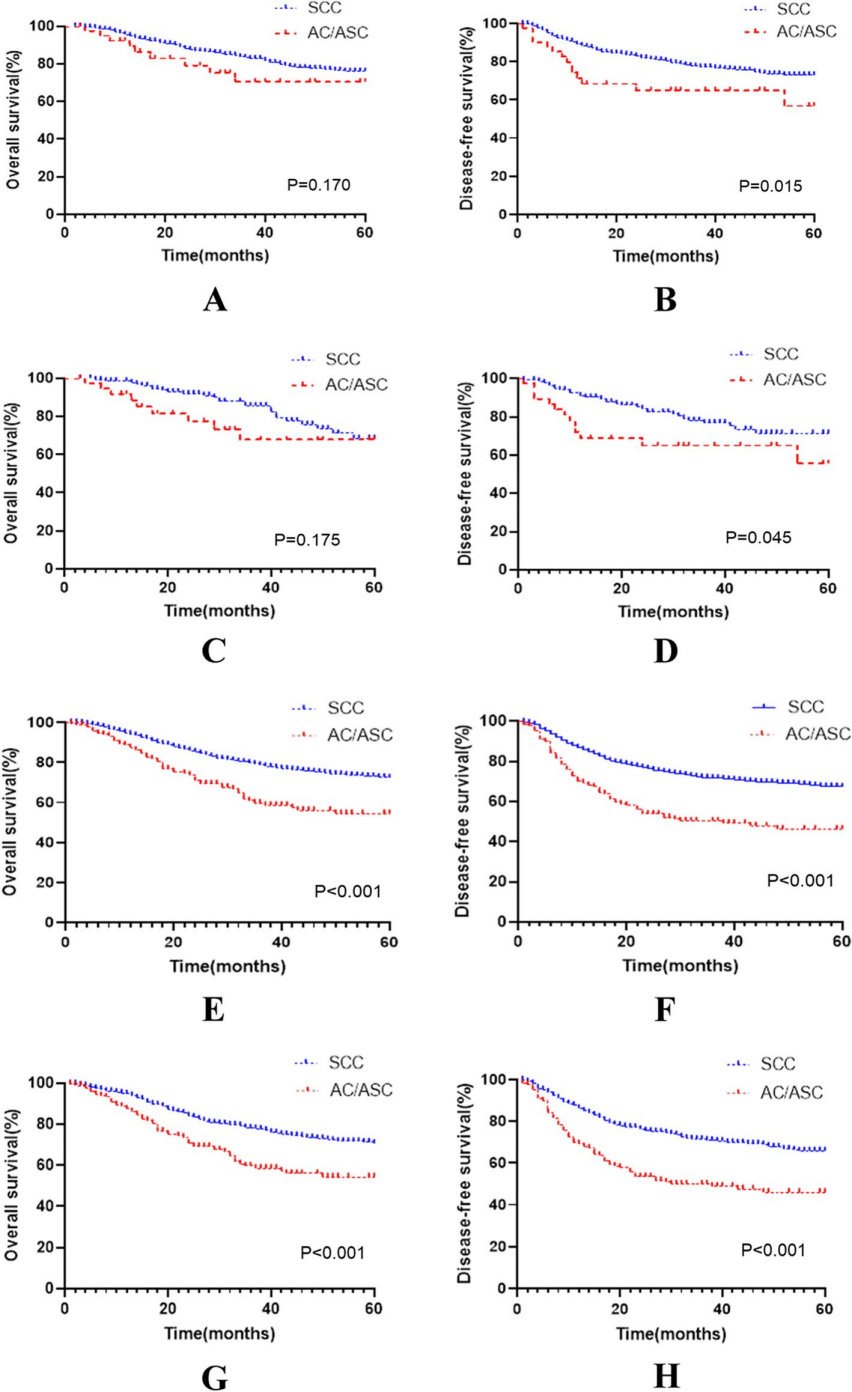
Survival curves before and after matching stage I to IV cervical cancer patients who met the study criteria. *Stage I to IIA2: before matching, panels A and B; after matching, panels C and D; Stage IIB to IV: before matching, panels E and F; after matching, panels G and H; *SCC* squamous cell carcinoma, *AC/ASC* adenocarcinoma/adenosquamous carcinoma

After matching, the median follow-up time was 36 months and 25 months. The number of deaths in the two groups within 5 years was 25 (17.48%) and 9 (23.68%). The 5-year OS of the two groups was 68.5% vs 67.8% (*P* = 0.075), and the DFS was 71.0% vs 55.7% (*P* = 0.045). Cox multivariate analysis showed that there was no significant difference in the risk of death between the two groups, but the risk of recurrence/death was higher in the AC/ASC group (Table 2, Fig. 3).

**Table 2:** Data on stage I-IIA2 patients before and after matching

Variables	Unmatched			Matched		
	**SCC (*n* = 807)**	**AC/ASC (*n* = 41)**	**p-value**	**SCC (*n* = 143)**	**AC/ASC (*n* = 38)**	**p-value**
**Age (years)**	56.25 ± 11.00	50.68 ± 13.27	0.002	53.94 ± 11.35	52.71 ± 11.36	0.556
**FIGO stage**			0.594			0.935
I	21 (2.6%)	1 (2.5%)		2 (1.4%)	1 (2.6%)	
II	8 (1.0%)	1 (2.5%)		2 (1.4%)	1 (2.6%)	
IB1	101 (12.5%)	6 (14.6%)		17 (11.8%)	6 (15.8%)	
IB2	79 (9.8%)	6 (14.6%)		19 (13.3%)	6 (15.8%)	
IIA	108 (13.4%)	3 (7.3%)		18 (12.6%)	3 (7.9%)	
IIA1	255 (31.6%)	10 (24.4%)		40 (28.0%)	9 (23.7%)	
IIA2	235 (29.1%)	14 (34.1)		45 (31.5%)	12 (31.6%)	
**Tumour size**			0.256			0.576
> 4 cm	319 (39.5%)	21 (51.2%)		62 (43.3%)	20 (52.6%)	
≤ 4 cm	325 (40.3%)	15 (36.6%)		56 (39.2%)	13 (34.2%)	
Unknown	163 (20.2%)	5 (12.2%)		25 (17.5%)	5 (13.2%)	

*FIGO* International Federation of Gynecology and Obstetrics, *SCC* squamous cell carcinoma, *AC/ASC* adenocarcinoma/adenosquamous carcinoma

### Differences in survival outcomes between the two groups before and after matching: patients with stage IIB and IV cervical cancer

In stage IIB-IV stage, the baseline was unbalanced between the SCC group (*n* = 4444) and the AC/ASC group (*n* = 174). After propensity score matching (1:4), 690 and 173 patients were included in each group. There was no significant difference in survival analysis between the two groups. Before matching, the median follow-up time was 31 months and 25 months, the number of deaths within 5 years was 872 (19.62%) and 59 (33.90%), the 5-year OS was 72.5% vs 67.1% (*P* < 0.001), and DFS was 67.1% vs 46.1% (*P* < 0.001), respectively. Cox multivariate analysis showed that the risk of death or recurrence/death was higher in the AC/ASC group (HR = 2.019, *p* < 0.001; HR = 2.131, *P* < 0.001).

After matching, the median follow-up times of the two groups were 31 months and 26 months. The number of deaths within 5 years was 145 (21.01%) and 59 (34.10%). The number of deaths or relapses was 192 (27.81%) and 70 (40.46%), respectively. The 5-year OS was 70.7% vs 54.3% (*P* < 0.001), and the DFS was 65.2% vs 45.8% (*P* < 0.001). Cox multivariate analysis showed that the risk of death or recurrence/death was higher in the AC/ASC group (HR = 1.940, *p* < 0.001; HR = 2.057, *P* < 0.001) (Table 3, Fig. 3).

**Table 3:** Data on patients with stage IIB-IV cervical cancer before and after matching

Variables	Unmatched			Matched		
	**SCC (*n* = 4444)**	**AC/ASC (*n* = 174)**	**p-value**	**SCC (*n* = 690)**	**AC/ASC (*n* = 173)**	**p-value**
Age (years)	55.67 ± 10.78	55.34 ± 10.66	0.694	55.82 ± 11.24	55.49 ± 10.51	0.721
FIGO stage			0.000			0.262
IIB	1845 (41.5%)	67 (38.5%)		308 (44.6%)	67 (38.7%)	
III	34 (0.8%)	4 (2.3%)		7 (1.0%)	4 (2.3%)	
IIIA	228 (5.1%)	6 (3.5%)		36 (5.2%)	6 (3.5%)	
IIIB	2142 (48.2%)	78 (44.8%)		295 (42.8%)	78 (45.1%)	
IV	61 (1.4%)	3 (1.7%)		10 (1.5%)	3 (1.7%)	
IVA	31 (0.7%)	4 (2.3%)		11 (1.6%)	4 (2.3%)	
IVB	103 (2.3%)	12 (6.9%)		23 (3.3%)	11 (6.4%)	
Tumour size			0.557			0.977
> 4 cm	2087 (47.0%)	75 (43.1%)		238 (34.5%)	61 (35.3%)	
≤ 4 cm	1429 (32.1%)	62 (35.6%)		305 (44.2%)	75 (43.3%)	
Unknown	928 (20.9%)	37 (21.3%)		147 (21.3%)	37 (21.4%)	

*FIGO* International Federation of Gynecology and Obstetrics, *SCC* squamous cell carcinoma, *AC/ASC* adenocarcinoma/adenosquamous carcinoma

## Discussion

In recent years, due to the popularity of cervical cancer screening, the global incidence of squamous cell carcinoma (SCC) has decreased significantly, from 90% in 1950–1960 to 75%, while the proportion of adenocarcinoma (AC) has increased year by year, from 5 to 20%- 25% [21]. The reason for this phenomenon may be that cytological screening of cervical cancer greatly reduces the incidence of squamous cell carcinoma, while adenocarcinoma is mostly endogenous, and most of the lesions are located at the inner mouth of the cervical canal, in which decreases the detection rate of adenocarcinoma by cytological screening [22, 23]. At present, there is no difference in treatment between cervical SCC and adenocarcinoma/adenosquamous carcinoma (AC/ASC), but the clinical features and prognosis of AC are different from those of SCC. Whether patients with different histological subtypes have different survival outcomes is still a controversial topic.

The previous literature has shown that in cases of radical radiotherapy and chemotherapy for cervical cancer, the oncological outcome of AC/ASC is worse than that of SCC. A study based on South Korea's national cancer incidence database showed that the survival rate of patients with cervical cancer was improved after simultaneous chemotherapy, but the survival rate of patients with AC was still lower than that of patients with SCC [9]. Meanwhile, Huang YT, Yokoi E and Hu K and other researchers founded the prognosis of AC/ASC patients is worse than that of SCC patients [10–12].The 5-year OS of SCC is 58.6%—85.2%, and the 5-year OS of AC/ASC is 26.7%—75.4%. However, some studies suggest that the oncological outcomes of the two are similar. For example, Rose PG et al. [13] found that the prognosis of AC/ASC after simultaneous radiotherapy and chemotherapy was similar to that of SCC. Katanyoo K et al. [14] thought that the histological type did not affect the survival outcome.

In this study, all cervical cancers were analysed according to the inclusion criteria, and it was found that the oncological outcome of the SCC group was better. After controlling for confounding factors by propensity score matching (1:4), the oncological outcome of the SCC group was still better. The 5-year OS was 72.2% vs 56.9%, *p* < 0.001, HR = 1.895; the DFS was 67.6% vs 47.8%, *p* < 0.001, HR = 2.054. Further analysis according to different stages revealed no significant difference in 5-year OS between the two groups before and after propensity score matching (1:4) in stage I-IIA2, but the 5-year DFS in the SCC group was higher than that in the AC/ASC group (before matching, OS: 76.1% vs 70.5%, *P* = 0.170; DFS: 72.8% vs 56.8%, *P* = 0.015, HR = 1.945, *P* = 0.018; after matching, OS: 68.5% vs 67.8%, *P* = 0.175; DFS: 71.0% vs 55.7%, *P* = 0.045, HR = 2.037, *P* = 0.033). For stage IIB-IV, the results both before and after matching suggested that the oncological outcome of the SCC group was better (before matching, OS: 72.5% vs 67.1%, *p* < 0.001, HR = 2.019; DFS: 67.1% vs 46.1%, *p* < 0.001, HR = 2.131; after matching, OS: 70.7% vs 54.3%, *P* < 0.001, HR = 1.940; DFS: 65.2% vs 45.8%, *P* < 0.001, HR = 2.057).

The results of this paper are contrary to the results of Rose PG and Katanyoo K [13, 14] and basically consistent with the results of Lee JY, Huang YT, Yokoi E and Hu K [9–12]. The reasons may be as follows: (1) The number of included cases differed across studies. Rose PG and Katanyoo K analysed 1671 cases (SCC 1489, AC/ASC 182) and 423 cases (SCC 282 and AC/ASC 141 cases), respectively, so the difference between groups may not be accurately reflected. However, Lee JY et al. analysed 80,766 cases (SCC 64,531, AC/ASC 7256). The sample size was so large that the result is similar to that in this paper. (2) We performed the Propensity score matching(1:4) while other studies didn’t used such statistical method. Due to the significant difference in baseline characteristics between the two groups, this study and Hu K use propensity score matching to exclude the influence of confounding factors, and the results are consistent. In addition, compared with the Korean database, which lacks important demographic variables, such as FIGO staging, socioeconomic status and recurrence time and distribution, this database has a large sample size, detailed case data and survival outcomes. Therefore, our conclusion is more credible.

In summary, the OS and DFS of cervical AC/ASC after radical radiotherapy and chemotherapy were worse than those of SCC; in stage I-IIA2, there was no significant difference in 5-year survival time, but patients with AC/ASC were more likely to relapse; and the oncological outcome of radical radiotherapy and chemotherapy of cervical AC/ASC in more advanced stage IIB-IV disease was worse than that of SCC. Therefore, for patients with stage I-IIA2 cervical AC/ASC, the principle of clinical treatment can rely on that of SCC. For patients with stage IIB-IV cervical AC/ASC, individualized treatment should be carried out according to the different conditions of the patients, but not completely in accordance with the treatment principles of SCC. It has been reported that simultaneous radiotherapy and chemotherapy combined with neoadjuvant chemotherapy is a promising method to improve the survival rate of patients with cervical AC [24], and the application of targeted drugs may provide new opportunities for the treatment of patients with cervical AC. In the future, more large-scale, high-quality prospective studies are needed to study the prognostic differences and treatment between AC/ASC and SCC to improve the clinical prognosis of patients.

Our study has the following limitations. Firstly, we collected the case with the treatment of radiotherapy + chemotherapy which also called "sequential therapy" in China. Due to various reasons, such as inconsistent economic development, different hospital levels, different patient compliance, etc., we have no way to carry out standard concurrent chemoradiotherapy.Therefore, radiotherapy + chemotherapy has specific application in diagnosis and treatment in China and the inclusion of them can more objectively and accurately reflect the diagnosis and treatment status of cervical cancer in China.Secondly, we performed stratification only for staging and did not compare the oncological outcomes of the two groups according to the treatment regimens. We plan to further explore to identify the optimal scheme in the future.

## Data Availability

The datasets used and/or analysed during the current study available from the corresponding author on reasonable request.

## Abbreviations

**SCC**: Squamous cell carcinoma
**AC**: Adenocarcinoma carcinoma
**ASC**: Adenosquamous carcinoma
**OS**: Overall survival
**DFS**: Disease-free survival
**FIGO**: International Federation of Gynecology and Obstetrics
**RWS**: Real-world study
**PACS**: Picture archiving and communication system
**RT**: Radiotherapy
**CCRT**: Concurrent chemoradiotherapy
